# Attitudes and Beliefs on Influenza Vaccination during the COVID-19 Pandemic: Results from a Representative Italian Survey

**DOI:** 10.3390/vaccines8040711

**Published:** 2020-11-30

**Authors:** Alexander Domnich, Maura Cambiaggi, Alessandro Vasco, Luca Maraniello, Filippo Ansaldi, Vincenzo Baldo, Paolo Bonanni, Giovanna Elisa Calabrò, Claudio Costantino, Chiara de Waure, Giovanni Gabutti, Vincenzo Restivo, Caterina Rizzo, Francesco Vitale, Riccardo Grassi

**Affiliations:** 1 Seqirus S.R.L., 53035 Monteriggioni, Italy; maura.cambiaggi@seqirus.com (M.C.); alessandro.vasco@seqirus.com (A.V.); 2 SWG S.p.A., 34133 Trieste, Italy; luca.maraniello@swg.it; 3 Azienda Ligure Sanitaria, 16121 Genoa, Italy; filippo.ansaldi@regione.liguria.it; 4 Planning and Prevention Unit, IRCCS San Martino Hospital, 16132 Genoa, Italy; 5 Department of Health Sciences, University of Genoa, 16132 Genoa, Italy; 6 Department of Cardiac Thoracic Vascular Sciences and Public Health, Public Health Section, University of Padua, 35131 Padua, Italy; vincenzo.baldo@unipd.it; 7 Department of Health Sciences, University of Florence, 50134 Florence, Italy; paolo.bonanni@unifi.it; 8 Section of Hygiene, University Department of Life Sciences and Public Health, Università Cattolica del Sacro Cuore, 00168 Rome, Italy; giovannaelisa.calabro@unicatt.it; 9 VIHTALI (Value in Health Technology and Academy for Leadership & Innovation), Spin-Off of Università Cattolica del Sacro Cuore, 00168 Rome, Italy; 10 Section of Hygiene, Department of Health Promotion Sciences, Maternal and Infantile Care, Internal Medicine and Medical Specialties “G. D’Alessandro”, University of Palermo, 90133 Palermo, Italy; claudio.costantino01@unipa.it (C.C.); vincenzo.restivo@unipa.it (V.R.); francesco.vitale@unipa.it (F.V.); 11 Department of Experimental Medicine, University of Perugia, 06132 Perugia, Italy; chiara.dewaure@unipg.it; 12 Department of Medical Sciences, University of Ferrara, 44121 Ferrara, Italy; giovanni.gabutti@unife.it; 13 Predictive and Preventive Medicine Research Unit, Multifactorial and Complex Disease Research Area, Bambino Gesù Children’s Hospital, IRCCS, 00165 Rome, Italy; caterina1.rizzo@opbg.net

**Keywords:** influenza, COVID-19, vaccine, survey, vaccine hesitancy, attitudes, Italy

## Abstract

The last 2019/20 northern hemisphere influenza season overlapped with the first wave of coronavirus disease 2019 (COVID-19) pandemic. Italy was the first western country where severe acute respiratory syndrome coronavirus 2 (SARS-CoV-2) spread to a significant extent. In this representative cross-sectional survey, we aimed to describe some opinions and attitudes of the Italian general population towards both influenza vaccination and the COVID-19 pandemic, and to identify potential modifiers of the decision-making process regarding the uptake of the 2020/21 influenza vaccine. A total of 2543 responses were analyzed. Although most (74.8%) participants valued influenza vaccination positively and declared that it should be mandatory, some misconceptions around influenza persist. The general practitioner was the main source of trusted information on influenza vaccines, while social networks were judged to be the least reliable. Younger and less affluent individuals, subjects not vaccinated in the previous season, and those living in smaller communities showed lower odds of receiving the 2020/21 season influenza vaccination. However, the COVID-19 pandemic may have positively influenced the propensity of being vaccinated against 2020/21 seasonal influenza. In order to increase influenza vaccination coverage rates multidisciplinary targeted interventions are needed. The role of general practitioners remains crucial in increasing influenza vaccine awareness and acceptance by effective counselling.

## 1. Introduction

Each year seasonal influenza carries an enormous health and socioeconomic burden [1,2]. For instance, it has been estimated [3] that on average worldwide, a total of 389,000 (294,000–518,000) respiratory deaths are attributable to influenza annually; most of these deaths occur in older adults. The most recent Italian estimates [4] suggest that 7027 and 24,981 excess influenza-attributable deaths occurred in the 2013/14 and 2016/17 seasons, respectively.

Vaccination represents the cornerstone public health intervention, most able to reduce the burden of seasonal influenza disease [1,5]. Nevertheless, vaccination coverage is still low/suboptimal in most countries [6,7] and well below the recommended rate of 75% [8] in most instances. Several populations [e.g., pregnant woman, older adults, young children, individuals ≥ 6 months of age affected by specific health conditions defined as increasing the risk of adverse influenza-related outcomes, and healthcare professionals (HCPs)] have been identified as principal targets of vaccination [1]; however, both recommendations and reimbursement policies differ significantly among countries [6,9]. In Italy, in the upcoming 2020/21 season influenza vaccination is recommended and fully reimbursed [10] for: (i) older adults aged ≥ 65 years (although in these latest recommendations those 60–64 years of age may also be eligible for vaccination free-of-charge); (ii) subjects aged 6 months to 64 years affected by predefined health conditions (e.g., chronic respiratory and cardiovascular pathologies, cancer and other forms of immunodeficiency, etc.); (iii) professionals employed in public services of primary interest (like HCPs, the police, and firefighters, etc.); (iv) professionals in close contact with animals (e.g., veterinarians, butchers, farmers, etc.); (v) other categories (e.g., blood donors, institutionalized subjects, etc.) [10].

Influenza vaccination uptake may depend on several factors that may be schematically categorized into: (i) structural social determinants (e.g., age, sex, socio-economic status, etc.); (ii) intermediary correlates (e.g., residential location, behavioral beliefs, social influences, vaccination history, perceived susceptibility, sources of information, etc.) and (iii) healthcare system-related factors (e.g., accessibility, affordability, knowledge and attitudes about vaccination, and physicians’ advice) [11]. However, a systematic review by Kohlhammer et al. [12] has underlined the crucial and primary role of physicians in promoting the annual uptake of influenza vaccine.

In several countries, the last 2019/20 northern hemisphere and 2020 southern hemisphere seasonal influenza epidemics overlapped (the so-called “first wave”) with a pandemic caused by the severe acute respiratory syndrome coronavirus 2 (SARS-CoV-2). Italy was the first western country where the SARS-CoV-2 virus that causes coronavirus disease 2019 (COVID-19) spread in a significant manner [13]. Indeed, an unprecedented increase in winter excess mortality (starting from week 10 of 2020) was observed in Italian older adults aged ≥ 65 years [14]. This overlap between seasonal influenza and COVID-19 was also associated with the so-called “epidemic of panic”; indeed, the number of web searches around the COVID-19 conducted in Italy increased dramatically and was well correlated with the officially reported incidence of the new coronavirus disease [15].

Both seasonal influenza and COVID-19 share several signs and symptoms, being both mainly respiratory pathologies and presenting a wide range of illnesses from asymptomatic/mild to severe disease and death [16]. It seems biologically implausible that an influenza vaccine can protect against SARS-CoV-2 that belongs to a different family of viruses. On the other hand, a recent ecological study [17] documented a moderate negative correlation between regional influenza vaccination coverage rates in the elderly and COVID-19 attributable deaths among 21 Italian regions and autonomous provinces. The authors have not excluded “a possible protective effect of influenza vaccination against COVID-19 acquisition or disease” that “might occur if the vaccine could stimulate sufficient trained innate immune memory that when another respiratory pathogen like SARS-CoV-2 occurred, the local lung immune system would be primed for a rapid response, and that could impact acquisition of SARS-CoV-2 or the COVID-19 disease course”. On the other hand, being an ecological study in nature, other possible explanations like the “healthy vaccine” bias and ecological fallacy could not be ruled out [17]. Similar results have been obtained by a more recent ecological study by Amato et al. [18]: in their multivariable analysis influenza vaccination coverage rates in Italian regions were independently and negatively associated with SARS-CoV-2 seroprevalence, hospitalizations for COVID-19 symptoms, admission to intensive care units, and deaths attributable to COVID-19. However, in the context of the COVID-19 pandemic, the major role of the 2020/21 influenza vaccination campaign will be important for two other main reasons: (i) to reduce the burden of seasonal influenza disease and the associated pressure on healthcare systems and (ii) to help HCPs to distinguish between different influenza-like illness (ILI) patients. For instance, in this regard the Italian region of Lazio has introduced a mandatory requirement for influenza vaccination for both older adults and HCPs [19]. Similarly, the regions of Sicily [20] and Calabria [21] have also introduced mandatory influenza vaccinations for all HCPs. It seems likely that the differential diagnosis between influenza and SARS-CoV-2 infections was the first driver of these public health measures.

The overall objective of this study was to assess and describe the beliefs, attitudes, and practices of a representative sample of Italian adults regarding influenza vaccination. A computer-assisted web interview (CAWI) was conducted at the height of the first wave of COVID-19 pandemic in Italy. The survey results may be useful for both national and regional stakeholders in formulating both (i) future seasonal influenza vaccination campaigns and (ii) pandemic preparedness plans.

## 2. Materials and Methods

### 2.1. Study Design and Participants

This cross-sectional study was reactively conceived and performed following the start of the COVID-19 epidemic (later re-classified as a pandemic) in Italy in order to understand and map current and future influenza vaccination-related beliefs, attitudes, and practices of the adult Italian population. The survey was conducted during a single week (18–24 May 2020) by SWG S.p.A. on behalf of Seqirus S.R.L. by CAWI. SWG S.p.A. is a specialized data science company with a community of over 60,000 registered and well-characterized individuals [22].

Considering both the exploratory nature of the survey and its novelty (no similar surveys had been identified during study conception), no formally established sample size could be determined a priori. On the basis of the previous SWG experience, we aimed to reach at least 2000 responses.

Subjects aged ≥ 18 years were sampled from the available SWG database in a two-stage probabilistic quota modality. At the first step, registered individuals were stratified into mutually exclusive clusters according to sex, age-class, geographical area, and size of the municipality of residence. Individuals in each cluster were then selected in a random manner in order to assure that the sample structure was representative of the adult Italian population as per the Italian National Institute of Statistics [23].

This non-interventional, opinion-based web survey was conducted in accordance with all applicable laws including the General Data Protection Regulation (GDPR) [24]. Participation was voluntary and SWG S.p.A takes full responsibility for protecting the data from individual interviewees.

### 2.2. Survey Instrument

The survey instrument was conceived by SWG S.p.A. and consisted of different items that may be semantically categorized into the following groups: (i) socioeconomic; (ii) previous influenza vaccine experience (including 2019/20 vaccine uptake); (iii) knowledge, beliefs and attitudes towards (influenza) vaccination in general and (iv) willingness to receive the 2020/21 influenza vaccine, also in relation to the COVID-19 pandemic. The survey also had several questions relating to some sentiments, attitudes, behaviors, and changes in daily activities during the first wave of COVID-19 pandemic. These latter items will not be discussed in the present paper. It was mandatory to reply to all the questions in the survey.

Socioeconomic characteristics included sex, age, municipality of residence, household pattern, type of employment, educational level [as measured by the international standard classification of education (ISCED)] [25], and perceived economic well-being. ISCED Level 1 corresponds to primary education, while ISCED Level 6 refers to PhD programs [25]. The detailed categorization rule used will be reported later in the text.

Participants were then asked about their previous uptake of influenza vaccine. The possible answers were: (i) I have never had a flu shot so far; (ii) I had a flu shot in the past but not in the previous 2019/20 season; (iii) I had a flu shot in the 2019/20 season for the first time; (iv) I had a flu shot both in the 2019/20 season and sometime in the past.

Attitudes, knowledge, and beliefs on influenza vaccination were assessed by seven four-point (strongly disagree, more disagree than agree, more agree than disagree, strongly agree) Likert-scale items. These seven items, reported later in the text, all had a semantically positive construct. However, in order to verify that sufficient attention was paid to the questionnaire, and therefore, that the responses received were valid and reliable, a semantically negatively-worded item was introduced as a control question (“vaccines are a fraud made up in order to profit the pharmaceutical companies”). Responses to the latter item were compared to responses on the positively-worded item “vaccines are crucial to guarantee public health and should be mandatory” and the (dis)agreement was quantified by means of Cohen’s κ and Spearman’s ρ coefficients. We also performed a sensitivity analysis by excluding respondents who provided opposite answers to these two items (see below).

The confidence in different sources of information on influenza vaccination were measured on a scale of one to ten, where one is the minimum level of confidence. The information sources assessed will be reported later in the text.

The willingness to receive the 2020/21 season influenza vaccination was assessed through a specific item with the following response options: “not for sure”, “probably not”, “I don’t know”, “probably yes” and “yes for sure”. Those participants likely not to receive the next 2020/21 season vaccination were further asked to indicate 1–2 main reasons explaining their decision. For this item the response options were: (i) influenza vaccines are made up only to profit the pharmaceutical companies; (ii) influenza vaccines do not work; (iii) I had a flu shot but got a fever/cold nevertheless; (iv) I am afraid of needles; (v) my doctor advised against; (vi) other reasons. Interviewees that confirmed that they are likely to receive the 2020/21 season influenza vaccine were asked to indicate the most probable month of administration (response options: September 2020, October 2020, November 2020, December 2020, January 2021 or “I don’t know”).

Providing that the 2020/21 influenza season is expected to be associated with some increase in demand, the participants were asked “If during the next season there is an influenza vaccine shortage, whose fault it would be?” Up to two responses could be selected among the following: (i) Ministry of Health; (ii) pharmaceutical companies; (iii) regional authorities; (iv) local health units; (v) pharmacies and (vi) other.

Finally, we asked the participants that will probably receive the 2020/21 season influenza vaccination “How the COVID-19 pandemic has impacted your willingness to receive the next flu shot?” The response categories were: (i) not at all, I would get a 2020/21 flu shot regardless (ii) a little; (iii) quite enough and (iv) greatly, if no COVID-19 pandemic occurred, I would have no intention to get a 2020/21 flu shot.

### 2.3. Statistical Analysis

For descriptive purposes categorical variables were expressed as proportions with 95% confidence intervals (CIs), while the continuous variables were expressed as means with standard deviations (SDs) or medians with interquartile ranges (IQRs), as appropriate.

In order to explore associations, as measured by the adjusted odds ratio (aOR), between not being willing to receive the next seasons influenza vaccination (individuals who declared “not for sure” and “probably not” were henceforth referred to as “vaccine reluctant subjects”) and a set of socioeconomic characteristics described above, multivariable logistic regression modelling was performed. Age was modeled as a continuous variable because during the preliminary analysis various age groupings displayed a substantial multi-collinearity with the variable of employment status. The base-case model considered all the participants, while in the sensitivity analysis we excluded those participants who answered oppositely to the control question (see above).

Finally, in order to verify the influence of the COVID-19 pandemic on intention to be vaccinated in the 2020/21 season, as compared with the previous 2019/20 vaccination, we performed an ordinal logistic regression analysis with the output expressed as the adjusted proportional odds ratio (pOR). The outcome was a four-level response pattern to the question “How has the COVID-19 pandemic impacted your willingness to receive the next flu shot?” (see above), while the independent variable of interest was vaccination status in the previous 2019/20 season. However, at a preliminary step (as shown by the confusion matrix) it emerged that the intermediate responses “a little” and “quite enough” could not be identified correctly. For this reason, these two response categories were grouped together in the category of “to some extent”.

All analyses were performed using R stats packages, version 4.0.0 [26].

## 3. Results

### 3.1. Sample Characteristics

A total of 9750 invitations were sent. A total of 2543 unique complete responses were received (a response rate of 26.1%) and included in the present analysis. For privacy reasons, we were not able to compare responders and non-responders from the point of view of principal socioeconomic characteristics. Regarding the responders, no major quality issues emerged.

The sociodemographic characteristics of the study participants are reported in Table 1. Briefly, males and females were nearly equally distributed, and their mean age was 46.7 (SD: 15.5) years. The sample was judged to be representative of the adult Italian population regarding principal sociodemographic characteristics (Table 1).

In the whole sample the self-reported influenza vaccination rate in the 2019/20 season was 27.4% (95% CI: 25.6–29.1%). A total of 35.9% (95% CI: 34.0–37.8%) reported at least one influenza vaccination in the past, but not in the 2019/20 season. By contrast, 56.0% (95% CI: 54.1–58.0%) of the interviewees had never received an influenza vaccine. As expected, in the previous season, self-reported influenza adherence increased with age, reaching a maximum in adults aged ≥ 65 years [57.3% (95% CI: 52.2–62.3%)], which resembles the officially reported estimate.

### 3.2. Attitudes Towards Influenza Vaccination

**Figure 1:**
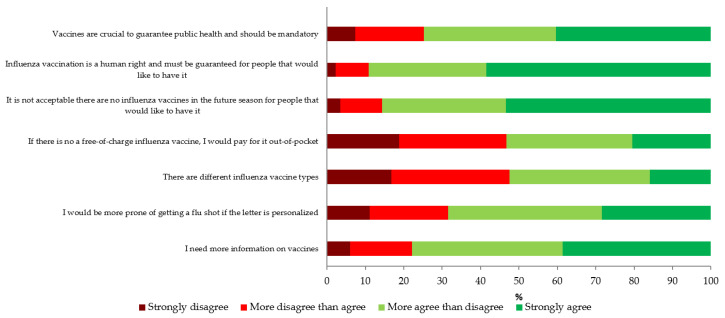
Beliefs, knowledge, and attitudes on influenza vaccination (*N* = 2543).

Figure 1 reports the detailed response pattern to the items relating to beliefs, knowledge, and attitudes on influenza vaccination. Overall, most people agreed to some extent that: vaccines are crucial for public health and should be mandatory [74.8% (95% CI: 73.0–76.4%)]; influenza vaccination is a human right and should be offered to everybody who wants to be immunized [89.2% (95% CI: 87.9–90.4%)]; it is not acceptable to not provide an influenza vaccine to everybody who would like to have it [85.6% (95% CI: 84.2–86.9%)]. On the other hand, only about a half of the participants agreed that there are different types of influenza vaccine [52.5% (95% CI: 50.5–54.4%)] and would personally pay if there was no free-of-charge vaccination offer [53.2% (95% CI: 51.3–55.2%)]. A total of 68.4% (95% CI: 66.5–70.2%) would be more prone to getting a more personalized influenza vaccine. Finally, about three fourths [77.8% (95% CI: 76.2–79.4%)] of interviewees would like to have more information on vaccines in general.

A control question stating that vaccines are a “fraud made up in order to profit the pharmaceutical industry” produced the following output: 73.7% (95% CI: 71.9–75.4%) of respondents disagreed, while the remaining 26.3% (95% CI: 24.6–28.1%) agreed to some extent. When compared with the response pattern on the question “vaccines are crucial to guarantee public health and should be mandatory” (this item had a reverse response category) no agreement was found (κ = −0.11; ρ = −0.49), confirming that most respondents paid sufficient attention to the survey. Indeed, only 11.0% (N = 281) of respondents provided the opposite answers to these two items.

**Figure 2:**
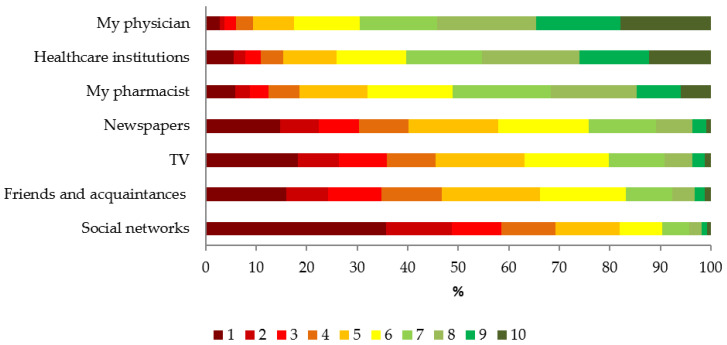
Perceived credibility of different sources of information on influenza vaccines (where 1 is not credible at all and 10 the most credible) (*N* = 2543).

When the participants were asked about the credibility of different information sources, the most credible source was considered to be the personal physician [median: 8 (IQR: 6–9)] followed by national/regional/local healthcare institutions [median: 7 (IQR: 5–9)] and personal pharmacists [median: 7 (IQR: 5–8)]. Friends [median: 5 (IQR: 3–6)], daily newspapers [median: 5 (IQR: 3–6)], TV [median: 5 (IQR: 2–6)], and social networks [median: 3 (IQR: 1–5)] were deemed to be less credible sources. The detailed response pattern is reported in Figure 2.

### 3.3. Willingness to Receive the 2020/21 Season Influenza Vaccination and Its Relationship to the COVID-19 Pandemic

Regarding the intention to receive an influenza vaccine in the 2020/21 season, a total of 18.3% (95% CI: 16.8–19.8%) and 23.6% (95% CI: 22.0–25.3%) responded “definitely no intention” and “probably not”, respectively. Conversely, a certain and likely willingness to be vaccinated was reported by 22.8% (95% CI: 21.2–24.4%) and 21.2% (95% CI: 19.6–22.8%) of interviewees, respectively. The remaining 14.1% (95% CI: 12.8–15.5%) replied “I don’t know”. The following reasons for not being willing to receive the 2020/21 influenza vaccine were more frequent: (i) influenza vaccines are made up only to profit the pharmaceutical companies [20.3% (95% CI: 18.2–22.6%)]; (ii) influenza vaccines do not work [17.7% (95% CI: 15.6–19.9%)]; (iii) I had a flu shot but got a fever/cold nevertheless [9.1% (95% CI: 7.6–10.8%)]; (iv) fear of needles [8.9% (95% CI: 7.4–10.6%)] and (v) my doctor advised against it [8.2% (95% CI: 6.8–9.9%)]. Most participants that were likely to comply with the next influenza vaccination campaign would receive a shot in the months of October [34.3% (95% CI: 31.9–36.8%)] or November [28.8% (95% CI: 26.5–31.2%)] 2020.

In the case of a shortage of influenza vaccines, survey participants believed (*N* = 3603 total responses since the participants could select one or two options) that this would primarily be the fault of: (i) the Ministry of Health [33.2% (95% CI: 31.6–34.7%)]; (ii) pharmaceutical companies [25.2% (95% CI: 23.8–26.7%)]; (iii) regional authorities [20.9% (95% CI: 19.6–22.3%)]; (iv) local health units [10.3% (95% CI: 9.3–11.3%)] and (v) pharmacies [2.9% (95% CI: 2.4–3.5%)]. The remaining 7.5% (95% CI: 6.7–8.4%) selected the option “other”.

We then explored socioeconomic factors that may be associated with reluctance to receive the vaccine for the 2020/21 season. Briefly, more reluctant individuals were observed among females, people living in smaller municipalities, and those with lower perceived economic well-being (Table 2). The mean age of reluctant individuals (43.4 versus 49.0 years) was also significantly (*t* = 9.46, *p* < 0.001) lower. However, the main determinant was the previous season’s influenza vaccination: the 2020/21 seasons reluctant individuals had only a 10.1% (versus 53.9%) coverage for the 2019/20 vaccination. The fully-adjusted logistic regression model established that the 2020/21 vaccine reluctant individuals were significantly younger, students, had about 10 times less probability of receiving the 2019/20 influenza vaccination, generally lived in smaller municipalities, and had a lower perceived income status. By contrast, people living alone or with at least one child were less likely to be reluctant to receive the next season’s vaccine (Table 2).

In the sensitivity analysis we excluded data from 281 subjects who provided the opposite response pattern to the control item (see above). The model output was consistent with the base-case analysis, although no dummy variable relative to the household pattern reached an *α* < 0.05 (Supplementary Material, Table S1).

Participants were finally asked if/how the COVID-19 pandemic changed their willingness to be vaccinated in the 2020/21 season. Most participants [33.5% (95% CI: 31.1–36.0%)] indicated no influence and that they would have the flu shot regardless. The replies “a little” and “quite enough” were received from 17.8% (95% CI: 15.9–19.8%) and 28.2% (95% CI: 25.9–30.6%) of participants, respectively. The remaining 20.4% (95% CI: 18.4–22.6%) were highly influenced and selected the answer “enormously, if no COVID-19 pandemic occurred, I would have no intention of getting the 2020/21 flu shot”.

These response patterns were then compared with the self-reported vaccination rate for the previous 2019/20 season. There was a negative association between the influence of the COVID-19 pandemic in Italy on the intention to receive the 2020/21 seasonal vaccine and the previous 2019/20 self-reported vaccination rate. In particular, about 89% of subjects that replied “nothing at all” were vaccinated in the previous season, while only 14% of those who replied “enormously” were immunized in the 2019/20 season. Multivariable ordinal logistic regression confirmed these observations: as compared with interviewees who received the previous 2019/20 season vaccine, those who did not receive the vaccine were significantly [pOR = 0.08 (95% CI: 0.06–0.10)] more likely to be influenced by the COVID-19 pandemic regarding their intention to receive the next season vaccine (Table 3). The model performed relatively well with an acceptable misclassification error of 37.8%.

## 4. Discussion

In the present study, we report results on some knowledge, beliefs, and attitudes of Italian adults on influenza vaccination. One of the main values of the present work is that, to our knowledge, it is among the first opinion surveys on influenza vaccination to be conducted in Italy during the first wave of COVID-19 pandemic; indeed, on 24 May (the last day of the survey), in Italy the cumulative number of COVID-19 cases and deaths was 229,858 and 32,785, respectively [27]. Furthermore, the present study has investigated how the COVID-19 pandemic may have impacted willingness to be vaccinated against influenza in the 2020/21 season. The results generated by the survey are corroborated by our nearly representative sample that could be judged to be sufficiently powered from the statistical point of view. Taken together, the survey findings may shed light on future regional and national vaccination policies, including pandemic preparedness plans. Principal findings and a comparison with previous research are discussed below.

The results obtained in the present study have several similar traits to a recent online survey conducted across six countries (United States, Canada, Israel, Japan, Spain and Switzerland) among caregivers of children aged 1–19 years [28]. The authors highlighted that the main predictors able to modify the decision-making process of caregivers regarding the vaccination of children for the next season were the perception of risk that the child will get COVID-19, the child’s vaccination status, and the caregiver’s influenza vaccination history [28].

Most study participants viewed the value of vaccines positively, and particularly valued influenza vaccines. However, only half of the interviewees would pay for an influenza vaccine themselves. In the context of the Italian Beveridge-like healthcare system, this finding underlines the importance of seasonal influenza vaccination as a priority and a universal public health intervention. Indeed, the value of influenza vaccination was recently (2017) recognized by the Italian Ministry of Health [29], including it among the so-called “essential levels of healthcare” (livelli essenziali di assistenza). On the other hand, currently, influenza vaccines in Italy are reimbursed only for older adults and some specific risk categories (as reported in the “Introduction” section) [10]. In this regard and on the basis of an exhaustive and multidisciplinary analysis of the available evidence, Italian scientific societies have been proposing for several years, a lowering of the free-of-charge vaccination offer from ≥65 to ≥60 years of age (or better still to ≥50 years) [30]. In this context, the role of public health physicians (according to their specific training) seems particularly prominent for establishing national, regional, and local influenza vaccination policies by leveraging different aspects of a given technology in a sound and systematic manner. The health technology assessment (HTA) procedure, which examines the (new) technology from 360 degrees, is probably the most suitable approach here. Indeed, four different Italian HTA reports specifically devoted to influenza vaccines have been published since 2015 [31,32,33,34] and then used [32,33] in the latest recommendations [35].

Only a half of the respondents agreed that the currently available influenza vaccines may be different. The field of influenza vaccination is continuously evolving, with several vaccine formulations having been made available during the last 2019/20 northern hemisphere season in the United States [9], Europe [36] and Italy [10]. Some of these formulations (the so-called “enhanced” vaccines, such as adjuvanted or high-dose formulations) have been specifically developed for older adults in order to mitigate some of the negative effects of immunosenescence on vaccine-induced immune responses [37,38]. Interestingly, in a recent survey by Boccalini et al. [39] only 10% of vaccinating physicians disagreed that the available influenza vaccines are the same. The most obvious reason for this discrepancy between our findings and those by Boccalini et al. [39] is the patient–HCP information asymmetry. Because most influenza vaccinations in Italy are administered by general practitioners (GPs) [40], the latter should have appropriate counseling skills in general [41] and be able to highlight the different available vaccine options and associated risk–benefit ratios in particular. A better understanding of the role of HCPs for alleviating asymmetric information among high-risk patients like the elderly, is important for the design of future health policy interventions and increasing vaccination coverage rates [42]. However, two Italian surveys regarding influenza seasons 2014/15 [43] and 2017/18 [39] found that only half of GPs had the possibility of choosing from among the available vaccine types. It has also been suggested [39] that the availability of explicit guidelines on the appropriateness of the use of individual influenza vaccine types could increase vaccine uptake in Italy. This fact underlines the importance of having clear Italian nationwide recommendations (similar, for example, to those issued in the United Kingdom [44]) and implementing interventions at the central level, aiming to homogenize the adoption of these recommendations at the regional/local level in order to smooth the well-known “jeopardized” character of the vaccination recommendations among the individual regions of Italy [45]. In turn, regional and local authorities should follow these nationwide guidelines and assure an equal and timely procurement of influenza vaccines. Indeed, in our survey when participants were asked who would be the main “guilty” party in the case of influenza vaccine shortages, 45.9%, 29.0% and 14.2% of interviewees declared the Ministry of Health, regional authorities, and local health units, respectively.

Two thirds of the study participants would be more likely to be immunized if the vaccines were more personalized. Public health professionals should have an important role to play in achieving personalized healthcare changes, including within the field of vaccinology. Public health values would be vital, in particular in disease prevention and health management [46]. In this regard, the criteria of appropriateness and sustainability seem particularly attractive: each citizen should receive the most appropriate vaccine in order to achieve adequate protection [47].

Among the possible reasons for vaccine refusal, those somehow linked to vaccine effectiveness should be considered in primis. In particular, 20.3% of respondents in the present survey declared that “influenza vaccines are made up only to profit the pharmaceutical companies” (i.e., their real value is low), while 17.7% and 9.1% believed that vaccines do not work or experienced an episode of fever/cold despite having received an influenza vaccine in the past, respectively. Generally speaking, there is no doubt that seasonal influenza vaccination represents value-for-money; this has been explicated in a systematic review by de Waure et al. [48]. Our findings are also coherent with a qualitative meta-analysis performed by Nowak et al. [49]; these authors found in several national surveys that a total of 20–33% of respondents indicated that “they did not believe the vaccine would protect them from influenza” [49]. Indeed, vaccination communication strategies are often mainly focused on safety aspects. However, particular virological and ecological features of the influenza virus [50] and several host-related characteristics able to modify the influenza vaccine-induced immune response [51] may determine a high variability in influenza vaccine effectiveness by year, age group, predominant virus (sub)type, degree of antigenic match [52], and possibly other factors. On the other side, acknowledgement that variable influenza vaccine effectiveness estimates may change the decision-making process by both vaccinating HCPs and patients, and the question of whether or not to communicate vaccine effectiveness data to patients is debatable. In this regard, Zhao et al. [53] have claimed that “vaccine effectiveness studies are designed to inform public health decisions rather than for individual decision-making” and “an individual’s decision to get vaccinated should be primarily informed by their risk of influenza illness and their risk of transmitting influenza to vulnerable people”. However, we believe that in the web 2.0 era, when the patient–HCP relationship paradigm has been changing continuously, a vaccinating HCP should inform the patient about expected levels of vaccine effectiveness. Although laypeople have some basic knowledge of influenza and generally perceive influenza as different/more severe than the common cold [49], we believe that HCPs should stress that only a part of ILI cases are due to influenza virus. Indeed, in Italy in the post-pandemic period only 23–38% of ILI cases were due to influenza [34].

Then, the results obtained confirmed that GPs are the most credible source of information on the annual influenza vaccination; this is in line with a systematic review by Kohlhammer et al. [12]. The counseling performed by vaccinating GPs is therefore crucial in promoting influenza vaccination and increasing coverage rates. Indeed, GPs being in close contact with their patients, are best placed to provide adequate vaccination counselling (according to existing recommendations, and including an assessment of benefit–risk ratio), to answer specific questions from their patients, to send informative materials, and to repeatedly offer vaccination [54]. This is the reason why, in the light of scientific advances in the field and the availability of new vaccines, GPs and other HCPs require continuous training in vaccinology [55]. On the other hand, 8.2% of respondents declared that they would not get an influenza vaccine because their physician advised against it. A similar estimate of 5.5% was obtained in a Portuguese study among high-risk adults [56]. As role models, physicians having been vaccinated themselves may serve to persuade their patients and help to further promote vaccination [57]. Indeed, Frank et al. [58] have demonstrated a 13.7% relative difference in influenza vaccination rates among patients whose physicians received the influenza vaccine versus those whose physicians had not. Despite the official recommendation and full reimbursement [10], in Italy the influenza vaccination coverage rate among HCPs is extremely low; a recent large multicenter study [59] has shown a coverage rate of only 14%. Moreover, among the different vaccines recommended to HCPs, seasonal influenza vaccines have been shown to have the lowest acceptance rate in a sample of Italian medical doctors [60].

Healthcare institutions and community pharmacists were the second and third most credible sources of information, respectively. The role of institutions is crucial in emanating clear messages and guidelines, these have been discussed previously. However, although the information provided by the Italian institutional bodies is generally of high quality and evidence-based, the web content of the institutions is may be suboptimal in terms of readability and/or comprehensibility; it is therefore advisable to improve the accessibility and user-friendliness parameters of the information provided [61].

Regarding pharmacists, in Italy community pharmacies may be seen as the most easily accessible health-related “place” for laypeople: individuals can enter a pharmacy without a previous appointment and receive professional advice (including vaccination counselling) immediately [62,63]. As described previously, in Italy, most vaccine doses are administered in GPs’ offices [40]. However, to our knowledge, a more diversified approach to the physical places where vaccine administration may take place has being discussed for years; the main goal would be to facilitate the administration of vaccine and thereby achieve the minimum recommended coverage rate of 75% [8]. Previous international experiences [64,65] found several advantages in the administration of influenza vaccines in drug stores. The current COVID-19 pandemic seems to accelerate some policy changes at the central level in Italy: according to a recent Chamber of Deputies agenda [66], it was proposed that influenza vaccines also be administered in community pharmacies, providing that the environment is hygienic, that medical doctors are present, and that other specific requirements are met. This proposal predicts an increase in influenza vaccination coverage rates [66]. Considering the increasing role of pharmacists in future influenza vaccination campaigns, we believe that pharmacists should be appropriately and continuously trained on immunization and influenza vaccines in particular. Indeed, an Italian study conducted in Umbria [67] reported that only 15.3% of pharmacists consulted scientific publications, while a total of 52.8% had not shown any interest in the influenza vaccine.

Other sources of information like traditional mass media and social networking websites in particular generated a significantly lower level of public confidence. We expected higher levels of confidence in social media platforms. Indeed, because internet access and usage in Italy is increasing continuously, the web has become a primary channel for the anti-vaccine movement [68,69]. However, in our study social networks were “voted” to be the least credible sources of information: only 18% of respondents attributed >5 points on the 1-to-10 rating scale. In summary, our study demonstrates that the role of social networks may sometimes be inflated and overestimated. Nevertheless, a more modern participatory healthcare model (e.g., where patients may bring information to the GP which has been downloaded from online resources) [69], most people still believe the GP and/or other HCPs/healthcare institutions to be the most reliable sources of information on seasonal influenza vaccination. On the other hand, from a whole population perspective, this 18% represents a large section of the population which may easily be influenced by misinformation provided by the anti-vaccine movement. The continuous development of Italian user-friendly and evidence-based online platforms (like vaccinarsi.org) able to reach the highest numbers of users possible is a priority [70,71].

In our sample survey, 41.6% of interviewees are likely not to have the 2020/21 season influenza vaccine; of these 18.3% will definitely not have the vaccine. Therefore, we investigated different socioeconomic factors than may determine influenza vaccine uptake in the next season—several significant associations were found. These results may be useful in prioritizing targeted health promotion interventions aiming to increase coverage rates. From the point of view of observed effect size, the first determinant was influenza vaccination during the previous season. A systematic review of the barriers to influenza vaccination intention and behavior issued by the World Health Organization (WHO) [72] reached similar conclusions: subjects who had been vaccinated in previous seasons showed higher vaccine uptake in all risk groups including HCPs, the elderly, pregnant women, and those with underlying chronic conditions. Second, we also documented that age was a significant structural social determinant of the uptake of the next seasons influenza vaccine, this finding was expected. Indeed, a systematic review by Nagata et al. [11] has underlined a similar pattern, independent from associated morbidity status. Third, in our study males were more prone to receive the 2020/21 influenza vaccination; however, the difference was only significant in the unadjusted analysis. This result is in line with the systematic review by Nagata et al. [11]: these authors established that although the bivariate analysis has often found a higher likelihood of men being vaccinated, the difference between sexes usually disappeared on multivariate regression analysis. Finally, we documented a meaningful association between the likelihood of future influenza vaccine administration and some patterns of urbanization and perceived economic well-being. Considering that these possible determinants are more controversial and that only a few Italian studies on these associations are available [11,72], it is important to discuss these results in more detail.

Italy may be considered to be a country of towns and villages [62]. In our study people living in smaller municipalities were generally more prone to refuse the next season’s influenza vaccination. Indeed, the highest effect size (aOR = 0.55) was observed in the comparison of municipalities with ≥250,000 versus < 5000 inhabitants. As shown in a systematic review by Crocker-Buque et al. [73] the level of urbanization may play a diversified role in the adoption of preventive public health measures such as influenza vaccination. First of all, geographical accessibility to different healthcare services may play role. Indeed, people living in areas of abundant supply (usually urbanized areas) may be subject to the so-called “supplier-induced demand” and therefore more prone to utilize these health-related services. In this regard, O’Leary et al. [74] have revealed that although attitudes to influenza vaccination were similar between urban and rural parents, vaccination coverage was 53% higher among children living in urban areas. Rural parents preferred influenza vaccination outside their provider’s office; the authors concluded that alternative venues for influenza vaccination in rural areas should be promoted [74]. Moreover, Italian rural areas are characterized by a relatively high density of the elderly [62], who are the main target population for influenza vaccination [10]. An older adult (especially the so-called “oldest old”) living in rural areas may have fewer transport options [62] and thus less probability of receiving a flu shot, as reported by a study conducted on vaccination service organization and accessibility in Italy [75]. However, we speculate that the observed difference could be also attributed to the different impact of the lockdown measures adopted during the COVID-19 outbreak. Indeed, when we asked “to what degree are you worried about the SARS-CoV-2 diffusion” (results not shown) there was a clear relationship (Kruskal–Wallis test: p = 0.026) between the median municipality size and sentiments (“nothing at all”: 37,834 inhabitants; “a little”: 40,661 inhabitants; “quite enough”: 44,401 inhabitants and “a lot”: 54,868 inhabitants). As proof, recently released preliminary results [76] of a representative survey conducted in the highly urbanized Italian region of Lombardy show a high impact of the pandemic on lifestyle habits and some behavioral risk factors of the adult population in this epicenter of COVID-19 disease in Italy.

In our study, less economically affluent respondents reported a lower propensity for the next season’s influenza vaccination, independent of educational level and other possible confounders. A recent systematic review by Vukovic et al. [77] revealed that individuals living in the most deprived areas had a lower influenza vaccination coverage, while the association between socio-economic indices and coverage was contrasting. Similar results have been obtained by another systematic review [78] which highlighted that this association may depend on the measures used to assess and define socio-economic status.

Finally, the data obtained suggest that the current COVID-19 pandemic may have impacted willingness to receive the 2020/21 influenza vaccine among people that had not received the previous 2019/20 season vaccine. This result is in line with the aforementioned online survey by Goldman et al. [28]. According to the regional tender allotments, the 2020/21 influenza season will see a significant increase in the procurement of vaccines, which is also linked to the mandatory recommendation for influenza vaccination in some at-risk groups in different Italian regions [19,20,21]. However, a lot of work must be done by the principal regional and national public health agencies in order to achieve this goal. As per previous experience during the 2009 influenza pandemic [79], despite the global threat, the uptake of the pandemic influenza vaccine in Italy was extremely low. In this regard, mathematical modelling [80] of the effects of the influenza vaccination campaign on the spread of CARS-CoV-2 and other respiratory pathogens suggests that increasing influenza vaccination rates may help to manage future respiratory outbreaks that overlap with the peak of seasonal influenza cases.

Despite a sufficiently large sample size and having a nearly representative (for the principal socioeconomic characteristics) sample of Italian adults, some study limitations must be taken into account. First of all, the cross-sectional nature of the present research did not allow for the comparison of changes in beliefs, attitudes, and practices toward seasonal influenza vaccination from before to after the start of the COVID-19 pandemic in Italy. This could be done only by indirect comparison with previously published studies.

Second, the present survey was reactively constructed and conducted during the COVID-19 pandemic in Italy and therefore no formal validation of the questionnaire could be performed in such a short time. Indeed, the formal validation of a questionnaire administered online may take several months. Likewise, we were not able to establish a sample size a priori, as per reasons indicated earlier.

Third, given the online nature of CAWI, we could not completely rule out the so-called “digital divide” bias and therefore its impact on the observed results. Indeed, our sample was composed of people able to connect to the web and therefore did not include Italians without an internet connection. However, we believe that this limitation had a low impact on the overall conclusions. Internet use in Italy is quite high with more than three quarters of Italian households having access [81]. Moreover, in our study respondents could answer the survey questions using mobile phones—this could potentially further diminish the digital divide bias.

Fourth, the obtained response rate of 26.1% may be seen as low/suboptimal. However, this relatively low proportion is an intrinsic limitation of most web-based surveys. Indeed, a systematic review by Poynton et al. [82] has established an average response rate in online surveys of 34.2% (SD = 22.6%). The response rate in our survey was therefore within this interval.

Finally, although the sample was representative from the point of view of principal sociodemographic characteristics, the observed whole population influenza vaccine coverage rate was higher than that officially reported for 2019/20 (27.4% versus 16.8%) [10]. On the other hand, vaccination coverage among adult participants ≥ 65 years old was very similar to that officially reported (57.3% versus 54.6%) [10]. The higher rates of vaccination coverage observed in people 18–64 years old could be ascribed to the social desirability bias. Nonetheless, we exclude the hypothesis that in our sample the participation rate was higher among people interested in influenza vaccination, due to the fact that the respondents did not know the interview topic before starting the questionnaire.

## 5. Conclusions

In conclusion, this survey demonstrates that the COVID-19 pandemic in Italy is likely to have some repercussions on the national level of influenza vaccination coverage for the season 2020/21. According the WHO guide on tailoring immunization programs [83], three objectives must be achieved: (i) identifying and prioritizing susceptible populations; (ii) diagnosing the demand- and supply-side barriers to vaccination; and (iii) designing evidence-informed responses. We believe that the results of our study addressed the first point above in depth by profiling a representative sample of Italian adults with regard to the 2020/21 influenza vaccination campaign. In particular, working age adults, individuals living in smaller municipalities, and those more economically disadvantaged should be the primary targets of an equally-based influenza vaccination campaign. Compulsory influenza vaccination programs, especially among those groups most at risk, may have a positive effect on both herd immunity [84] and overall winter excess mortality [17].

## Figures and Tables

**Table 1:** Sociodemographic characteristics of the study participants (*N* = 2543).

Variable	Level	% (*N*)	95% CI
Sex	Male	54.5 (1386)	52.5–56.5
	Female	45.5 (1157)	43.5–47.5
Age, years	18–24	9.3 (236)	8.2–10.5
	25–34	16.5 (420)	15.1–18.2
	35–44	18.7 (476)	17.2–20.3
	45–54	23.0 (586)	21.4–24.7
	55–64	17.1 (436)	15.7–18.7
	65–74	12.4 (315)	11.1–13.7
	≥75	2.9 (74)	2.3–3.6
Geographic area	North-East	19.6 (499)	18.1–21.2
	North-West	28.1 (714)	26.3–29.9
	Center	20.1 (512)	18.6–21.7
	South	21.9 (557)	20.3–23.6
	Islands	10.3 (261)	9.1–11.5
Urbanization pattern, *N* of inhabitants	<5000	11.4 (291)	10.2–12.7
	5000–9999	11.1 (283)	9.9–12.4
	10,000–29,999	18.5 (470)	17.0–20.0
	30,000–99,999	22.1 (561)	20.5–23.7
	100,000–249,999	11.1 (283)	9.9–12.4
	≥250,000	25.8 (655)	24.1–27.5
Employment status	Unemployed	8.5 (215)	7.4–9.6
	Student	9.2 (235)	8.1–10.4
	Housekeeper	5.6 (142)	4.7–6.5
	Occasionally employed	7.6 (192)	6.6–8.6
	Permanently employed	51.6 (1313)	49.7–53.6
	Retired	15.8 (402)	14.4–17.2
	Other	1.7 (44)	1.3–2.3
Household pattern	Living alone	11.0 (280)	9.8–12.3
	Living with a spouse/partner	54.6 (1388)	52.6–56.5
	Living with ≥ 1 child	39.8 (1013)	37.9–41.8
	Living with ≥ 1 older adult	15.8 (114)	3.7–5.4
Perceived economic well-being	Low	3.3 (84)	2.6–4.1
	Lower than average	10.3 (263)	9.2–11.6
	Average	38.9 (989)	37.0–40.8
	Higher than average	44.5 (1131)	42.5–46.4
	High	3.0 (76)	2.4–3.7
ISCED educational level	1	0.7 (19)	0.5–1.2
	2	7.1 (180)	6.1–8.1
	3/4	41.7 (1061)	39.8–43.7
	5	48.8 (1240)	46.8–50.7
	6	1.7 (43)	1.2–2.3

**Table 2:** Multivariable logistic regression analysis on the association between socioeconomic factors and reluctance to receive the 2020/21 influenza vaccine (*N* = 2543).

Variable	Level	% (95% CI) of Subjects that Will Unlikely Receive the 2020/21 Flu Shot	aOR (95% CI)	*p*
Sex	Male	38.7 (36.2–41.4)	Ref	–
	Female	45.7 (42.8–48.6)	0.94 (0.78–1.14)	0.55
Age, years	1-year increase	–	0.98 (0.97–0.99)	<0.001
2019/20 vaccination	Yes	10.1 (7.9–12.5)	Ref	–
	No	53.9 (51.6–56.2)	0.10 (0.08–0.13)	<0.001
Geographic area	North-East	40.1 (35.8–44.5)	Ref	–
	North-West	44.8 (41.1–48.6)	1.13 (0.86–1.49)	0.36
	Center	42.2 (37.9–46.6)	1.02 (0.76–1.38)	0.87
	South	38.4 (34.4–42.6)	0.79 (0.59–1.06)	0.11
	Islands	44.4 (38.3–50.7)	1.08 (0.76–1.52)	0.67
Urbanization pattern, *N* of inhabitants	<5000	50.5 (44.6–56.4)	Ref	–
	5000–9999	48.1 (42.1–54.0)	0.87 (0.61–1.27)	0.48
	10,000–29,999	40.0 (35.5–44.6)	0.61 (0.44–0.85)	0.004
	30,000–99,999	41.5 (37.4–45.7)	0.70 (0.51–0.97)	0.031
	100,000–249,999	41.7 (35.9–47.7)	0.77 (0.52–1.12)	0.17
	≥250,000	37.3 (33.5–41.1)	0.55 (0.40–0.76)	<0.001
Employment status	Permanently employed	45.7 (43.0–48.4)	Ref	–
	Occasionally employed	45.8 (38.6–53.2)	0.87 (0.62–1.23)	0.43
	Student	43.4 (37.0–50.0)	0.56 (0.38–0.81)	0.002
	Housekeeper	42.3 (34.0–50.8)	0.90 (0.60–1.37)	0.65
	Unemployed	53.5 (46.6–60.3)	1.34 (0.95–1.88)	0.091
	Retired	20.4 (16.6–24.7)	0.80 (0.55–1.15)	0.23
	Other	43.2 (28.3–59.0)	0.77 (0.40–1.51)	0.65
Household pattern	Living alone	45.4 (39.4–51.4)	1.41 (1.01–1.99)	0.046
	Living with a spouse/partner	40.2 (37.6–42.8)	1.04 (0.83–1.29)	0.74
	Living with ≥ 1 child	43.6 (40.6–46.8)	1.28 (1.05–1.58)	0.017
	Living with ≥ 1 older adult	43.0 (33.7–52.6)	1.11 (0.72–1.73)	0.63
Perceived economic well-being	Low	58.3 (47.1–69.0)	Ref	–
	Lower than average	48.3 (42.1–54.5)	0.64 (0.36–1.13)	0.12
	Average	41.8 (38.7–44.9)	0.51 (0.30–0.86)	0.011
	Higher than average	40.1 (37.2–43.0)	0.48 (0.28–0.82)	0.007
	High	31.6 (21.4–43.3)	0.34 (0.16–0.70)	0.004
ISCED educational level	1	26.3 (9.1–51.2)	Ref	–
	2	37.8 (30.7–45.3)	0.97 (0.29–3.24)	0.97
	3/4	43.6 (40.6–46.7)	1.12 (0.35–3.60)	0.84
	5	41.5 (38.7–44.3)	0.93 (0.29–2.98)	0.90
	6	37.2 (23.0–53.3)	1.15 (0.29–4.56)	0.84

**Table 3:** Influence of coronavirus disease 2019 (COVID-19) pandemic on the intention of being vaccinated in the 2020/21 season, as compared with the previous 2019/20 vaccination (season 2019/20) (*N* = 1477).

Influence of COVID-19 Pandemic on the Intention to Receive the 2020/21 Influenza Vaccination	Vaccination in 2019/20 Season, % (95% CI)		
	% (*N*)	95% CI	pOR (95% CI) ^a^
Nothing at all, I would get a 2020/21 flu shot regardless	83.2 (495)	79.6–86.4	0.08 (0.06–0.10)
To some extent	25.3 (680)	22.1–28.7	
Enormously, if no COVID-19 pandemic occurred, I would have no intention to get a 2020/21 flu shot	13.9 (302)	10.2–18.3	

^a^*p* < 0.001; adjusted for sex, age, geographic area, urbanization pattern, employment status, household pattern, perceived income and educational level.

## Supplementary Materials

The following are available online at https://www.mdpi.com/2076-393X/8/4/711/s1, Table S1: multivariable logistic regression analysis on the association between socioeconomic factors and reluctance to receive the 2020/21 influenza vaccine: sensitivity analysis (*N* = 2262).

## References

[1] World Health Organization (WHO) (2012). Vaccines against influenza WHO position paper—November 2012. Wkly. Epidemiol. Rec..

[2] GBD 2017 Influenza Collaborators (2019). Mortality, morbidity, and hospitalisations due to influenza lower respiratory tract infections, 2017: An analysis for the Global Burden of Disease Study 2017. Lancet Respir. Med..

[3] Paget J., Spreeuwenberg P., Charu V., Taylor R.J., Iuliano A.D., Bresee J., Simonsen L., Viboud C. (2019). Global Seasonal Influenza-associated Mortality Collaborator Network and GLaMOR Collaborating Teams. Global mortality associated with seasonal influenza epidemics: New burden estimates and predictors from the GLaMOR Project. J. Glob. Health.

[4] Rosano A., Bella A., Gesualdo F., Acampora A., Pezzotti P., Marchetti S., Ricciardi W., Rizzo C. (2019). Investigating the impact of influenza on excess mortality in all ages in Italy during recent seasons (2013/14–2016/17 seasons). Int. J. Infect. Dis..

[5] de Lusignan S., Correa A., Ellis J., Pebody R. (2016). Influenza vaccination: In the UK and across Europe. Br. J. Gen. Pract..

[6] European Centre for Disease Prevention and Control (ECDC) Seasonal Influenza Vaccination and Antiviral Use in EU/EEA Member States. https://www.ecdc.europa.eu/sites/default/files/documents/seasonal-influenza-antiviral-use-2018.pdf.

[7] Organization for Economic Co-Operation and Development (OECD) Influenza Vaccination Rates (Indicator). https://data.oecd.org/healthcare/influenza-vaccination-rates.htm.

[8] World Health Organization (WHO) Evaluation of Seasonal Influenza Vaccination Policies and Coverage in the WHO European Region. https://www.euro.who.int/__data/assets/pdf_file/0003/241644/Evaluation-of-seasonal-influenza-vaccination-policies-and-coverage-in-the-WHO-European-Region.pdf.

[9] Grohskopf L.A., Alyanak E., Broder K.R., Walter E.B., Fry A.M., Jernigan D.B. (2019). Prevention and control of seasonal influenza with vaccines: Recommendations of the Advisory Committee on Immunization Practices—United States, 2019–2020 influenza season. MMWR Recomm. Rep..

[10] Italian Ministry of Health Prevention and Control of Influenza: Recommendations for Season 2020–2021. http://www.trovanorme.salute.gov.it/norme/renderNormsanPdf?anno=2020&codLeg=74451&parte=1%20&serie=null.

[11] Nagata J.M., Hernández-Ramos I., Kurup A.S., Albrecht D., Vivas-Torrealba C., Franco-Paredes C. (2013). Social determinants of health and seasonal influenza vaccination in adults ≥65 years: A systematic review of qualitative and quantitative data. BMC Public Health.

[12] Kohlhammer Y., Schnoor M., Schwartz M., Raspe H., Schäfer T. (2007). Determinants of influenza and pneumococcal vaccination in elderly people: A systematic review. Public Health.

[13] Sarli L. (2020). The pandemic from COVID 19: A lesson that we must not forget. Acta Biomed..

[14] Italian National Institute of Health FluNews Italia. https://www.epicentro.iss.it/influenza/flunews.

[15] Lippi G., Mattiuzzi C., Cervellin G. (2020). Google search volume predicts the emergence of COVID-19 outbreaks: Google Trends and COVID-19 outbreak. Acta Biomed..

[16] World Health Organization (WHO) Q&A: Influenza and COVID-19—Similarities and Differences. https://www.who.int/emergencies/diseases/novel-coronavirus-2019/question-and-answers-hub/q-a-detail/q-a-similarities-and-differences-covid-19-and-influenza?gclid=CjwKCAjwmMX4BRAAEiwA-zM4JuVJiMhiqtIBrtEvZRgi3Gd7aDMbfSluWM-e-Su_hNuiLUkR-O2-FRoCSykQAvD_BwE.

[17] Marín-Hernández D., Schwartz R.E., Nixon D.F. (2020). Epidemiological evidence for association between higher influenza vaccine uptake in the elderly and lower COVID-19 deaths in Italy. J. Med. Virol..

[18] Amato M., Werba J.P., Frigerio B., Coggi D., Sansaro D., Ravani A., Ferrante P., Veglia F., Tremoli E., Baldassarre D. (2020). Relationship between influenza vaccination coverage rate and COVID-19 outbreak: An Italian ecological study. Vaccines (Basel).

[19] Region of Lazio Mandatory Order for Influenza and Pneumococcal Vaccination. http://www.regione.lazio.it/rl/coronavirus/ordinanza-per-vaccinazione-antinfluenzale-e-anti-pneumococcica-obbligatoria/.

[20] Region of Sicily Influenza Vaccination Campaign 2020/2021: Involvement of GPs and Pediatricians. https://www.vaccinarsinsicilia.org/assets/uploads/files/da-n.-743.pdf.

[21] Region of Calabria Decree of the President of the Region of Calabria N 47 of 27th May 2020. https://portale.regione.calabria.it/website/portalmedia/2020-05/ORDINANZA-DEL-PRESIDENTE-DELLA-REGIONE-N.47-DEL-27-MAGGIO-2020.pdf.

[22] SWG S.p.A. Activity. https://www.swg.it/activity.

[23] Italian National Institute of Statistics (ISTAT) Demography in Numbers. http://demo.istat.it/.

[24] European Union (EU) Regulation (EU) 2016/679 of the European Parliament and of the Council of 27 April 2016 on the Protection of Natural Persons with Regard to the Processing of Personal Data and on the Free Movement of Such Data, and Repealing Directive 95/46/EC (General Data Protection Regulation). https://eur-lex.europa.eu/eli/reg/2016/679/oj.

[25] Italian National Institute of Statistics (ISTAT) ISTAT Classification of the Italian Education Qualifications. https://www.istat.it/it/files/2011/01/Classificazione-titoli-studio-28_ott_2005-nota_metodologica.pdf.

[26] R Core Team R: A Language and Environment for Statistical Computing. http://www.R-project.org/.

[27] Italian Ministry of Health A New Coronavirus. http://www.salute.gov.it/portale/nuovocoronavirus/dettaglioNotizieNuovoCoronavirus.jsp?lingua=italiano&menu=notizie&p=dalministero&id=4807.

[28] Goldman R.D., McGregor S., Marneni S.R., Katsuta T., Griffiths M.A., Hall J.E., Seiler M., Klein E.J., Cotanda C.P., Gelernter R. (2020). Willingness to vaccinate children against influenza after the COVID-19 pandemic. J. Pediatr..

[29] Italian Ministry of Health Definition and Update of the Essential Levels of Healthcare. http://www.trovanorme.salute.gov.it/norme/dettaglioAtto?id=58669&completo=false.

[30] Bonanni P., Gasparini R., Greco D., Mennini F.S., Rossi A., Signorelli C. Lowering the Age of Influenza Vaccine Recommendation to 60 Years: A Choice for the Public Health and Economy. http://www.societaitalianaigiene.org/site/new/images/docs/gdl/vaccini/201360enni.pdf.

[31] Kheiraoui F., Cadeddu C., Quaranta G., Poscia A., Raponi M., de Waure C., Boccalini S., Pellegrino E., Bellini I., Pieri L. (2015). Health technology assessment of the quadrivalent influenza vaccine FLU-QIV (Fluarix Tetra^®^). Quad. Ital. J. Public Health.

[32] Di Pietro M.L., Poscia A., Specchia M.L., de Waure C., Zace D., Gasparini R., Amicizia D., Lai P.L., Panatto D., Arata L. (2017). Health technology assessment of the adjuvanted influenza vaccine in the Italian elderly population. Quad. Ital. J. Public Health.

[33] Boccalini S., Bechini A., Innocenti M., Sartor G., Manzi F., Bonanni P., Panatto D., Lai P.L., Zangrillo F., Rizzitelli E. (2018). The universal influenza vaccination in children with Vaxigrip Tetra^®^ in Italy: An evaluation of health technology assessment. J. Prev. Med. Hyg..

[34] Calabrò G.E., Boccalini S., Del Ricco M., Ninci A., Manzi F., Bechini A., Bonanni P., Panatto D., Lai P.L., Amicizia D. (2019). Health technology assessment of the quadrivalent cell culture-derived influenza vaccine Fluacelvax Tetra. Quad. Ital. J. Public Health.

[35] The Italian Lifetime Vaccination Calendar, 4th Edition 2019. http://www.igienistionline.it/docs/2019/21cvplv.pdf.

[36] European Centre for Disease Prevention and Control (ECDC) Types of Seasonal Influenza Vaccine. https://www.ecdc.europa.eu/en/seasonal-influenza/prevention-and-control/vaccines/types-of-seasonal-influenza-vaccine.

[37] Nicolay U., Heijnen E., Nacci P., Patriarca P.A., Leav B. (2019). Immunogenicity of aIIV3, MF59-adjuvanted seasonal trivalent influenza vaccine, in older adults ≥65 years of age: Meta-analysis of cumulative clinical experience. Int. J. Infect. Dis..

[38] Samson S.I., Leventhal P.S., Salamand C., Meng Y., Seet B.T., Landolfi V., Greenberg D., Hollingsworth R. (2019). Immunogenicity of high-dose trivalent inactivated influenza vaccine: A systematic review and meta-analysis. Expert Rev. Vaccines.

[39] Boccalini S., Tacconi F.M., Lai P.L., Bechini A., Bonanni P., Panatto D. (2019). Appropriateness and preferential use of different seasonal influenza vaccines: A pilot study on the opinion of vaccinating physicians in Italy. Vaccine.

[40] European Centre for Disease Prevention and Control (ECDC) The Organization and Delivery of Vaccination Services in the European Union. https://ec.europa.eu/health/sites/health/files/vaccination/docs/2018_vaccine_services_en.pdf.

[41] Costantino C., Vitale F. (2016). Influenza vaccination in high-risk groups: A revision of existing guidelines and rationale for an evidence-based preventive strategy. J. Prev. Med. Hyg..

[42] Maurer J. (2009). Who has a clue to preventing the flu? Unravelling supply and demand effects on the take-up of influenza vaccinations. J. Health Econ..

[43] Levi M., Bonanni P., Biffino M., Conversano M., Corongiu M., Morato P., Maio T. (2018). Influenza vaccination 2014-2015: Results of a survey conducted among general practitioners in Italy. Hum. Vaccin. Immunother..

[44] Nation Healthcare Service (NHS) of England The National Flu Immunisation Programme 2020/21. https://www.england.nhs.uk/wp-content/uploads/2020/05/national-flu-immunisation-programme-2020-2021.pdf.

[45] Barbieri M., Capri S., Waure C., Boccalini S., Panatto D. (2017). Age- and risk-related appropriateness of the use of available influenza vaccines in the Italian elderly population is advantageous: Results from a budget impact analysis. J. Prev. Med. Hyg..

[46] Ricciardi W., Boccia S. (2017). New challenges of public health: Bringing the future of personalised healthcare into focus. Eur. J. Public Health.

[47] Calabrò G.E., Specchia M.L., Boccalini S., Panatto D., Rizzo C., Merler S., Ferriero A.M., Di Pietro M.L., Bonanni P., de Waure C. (2020). Strengthening the evidence-based approach to guiding effective influenza vaccination policies. Vaccines (Basel).

[48] de Waure C., Veneziano M.A., Cadeddu C., Capizzi S., Specchia M.L., Capri S., Ricciardi W. (2012). Economic value of influenza vaccination. Hum. Vaccin. Immunother..

[49] Nowak G.J., Sheedy K., Bursey K., Smith T.M., Basket M. (2015). Promoting influenza vaccination: Insights from a qualitative meta-analysis of 14 years of influenza-related communications research by U.S. Centers for Disease Control and Prevention (CDC). Vaccine.

[50] Petrova V.N., Russell C.A. (2018). The evolution of seasonal influenza viruses. Nat. Rev. Microbiol..

[51] Domnich A., Manini I., Calabrò G.E., de Waure C., Montomoli E. (2019). Mapping host-related correlates of influenza vaccine-induced immune response: An umbrella review of the available systematic reviews and meta-analyses. Vaccines (Basel).

[52] Belongia E.A., Simpson M.D., King J.P., Sundaram M.E., Kelley N.S., Osterholm M.T., McLean H.Q. (2016). Variable influenza vaccine effectiveness by subtype: A systematic review and meta-analysis of test-negative design studies. Lancet Infect. Dis..

[53] Zhao L., Stirling R., Young K. (2019). Should individuals use influenza vaccine effectiveness studies to inform their decision to get vaccinated?. Can. Commun. Dis. Rep..

[54] Kassianos G., Blank P., Falup-Pecurariu O., Kuchar E., Kyncl J., De Lejarazu R.O., Nitsch-Osuch A., van Essen G.A. (2016). Influenza vaccination: Key facts for general practitioners in Europe: A synthesis by European experts based on national guidelines and best practices in the United Kingdom and the Netherlands. Drugs Context.

[55] Public Health England National Minimum Standards and Core Curriculum for Immunisation Training for Registered Healthcare Practitioners. https://assets.publishing.service.gov.uk/government/uploads/system/uploads/attachment_data/file/679824/Training_standards_and_core_curriculum_immunisation.pdf.

[56] Santos A.J., Kislaya I., Machado A., Nunes B. (2017). Beliefs and attitudes towards the influenza vaccine in high-risk individuals. Epidemiol. Infect..

[57] Holt D., Bouder F., Elemuwa C., Gaedicke G., Khamesipour A., Kisler B., Kochhar S., Kutalek R., Maurer W., Obermeier P. (2016). The importance of the patient voice in vaccination and vaccine safety-are we listening?. Clin. Microbiol. Infect..

[58] Frank E., Dresner Y., Shani M., Vinker S. (2013). The association between physicians’ and patients’ preventive health practices. CMAJ.

[59] Genovese C., Picerno I.A.M., Trimarchi G., Cannavò G., Egitto G., Cosenza B., Merlina V., Icardi G., Panatto D., Amicizia D. (2019). Vaccination coverage in healthcare workers: A multicenter cross-sectional study in Italy. J. Prev. Med. Hyg..

[60] Riccò M., Cattani S., Casagranda F., Gualerzi G., Signorelli C. (2017). Knowledge, attitudes, beliefs and practices of occupational physicians towards vaccinations of health care workers: A cross sectional pilot study in North-Eastern Italy. Int. J. Occup. Med. Environ. Health.

[61] Panatto D., Amicizia D., Arata L., Lai P.L., Gasparini R. (2018). A comprehensive analysis of Italian web pages mentioning squalene-based influenza vaccine adjuvants reveals a high prevalence of misinformation. Hum. Vaccin. Immunother..

[62] Domnich A., Arata L., Amicizia D., Signori A., Gasparini R., Panatto D. (2016). Assessing spatial inequalities in accessing community pharmacies: A mixed geographically weighted approach. Geospat. Health.

[63] Scarpitta F., Restivo V., Bono C.M., Sannasardo C.E., Vella C., Ventura G., Bono S., Palmeri S., Caracci F., Casuccio A. (2019). The role of the Community Pharmacist in promoting vaccinations among general population according to the National Vaccination Plan 2017–2019: Results from a survey in Sicily, Italy. Ann. Ig..

[64] Poulose S., Cheriyan E., Cheriyan R., Weeratunga D., Adham M. (2015). Pharmacist-administered influenza vaccine in a community pharmacy: A patient experience survey. Can. Pharm. J..

[65] Kirkdale C.L., Nebout G., Megerlin F., Thornley T. (2017). Benefits of pharmacist-led flu vaccination services in community pharmacy. Ann. Pharm. Fr..

[66] Mondelli Agenda. http://www.quotidianosanita.it/allegati/allegato177299.pdf.

[67] Gianfredi V., Nucci D., Salvatori T., Orlacchio F., Villarini M., Moretti M., PErCEIVE IN UMBRIA STUDY GROUP (2018). “PErCEIVE in Umbria”: Evaluation of anti-influenza vaccination’s perception among Umbrian pharmacists. J. Prev. Med. Hyg..

[68] Kata A. (2012). Anti-vaccine activists, Web 2.0, and the postmodern paradigm—An overview of tactics and tropes used online by the anti-vaccination movement. Vaccine.

[69] Amicizia D., Domnich A., Gasparini R., Bragazzi N.L., Lai P.L., Panatto D. (2013). An overview of current and potential use of information and communication technologies for immunization promotion among adolescents. Hum. Vaccin. Immunother..

[70] Ferro A., Odone A., Siddu A., Colucci M., Anello P., Longone M., Marcon E., Castiglia P., Bonanni P., Signorelli C. (2015). Monitoring the web to support vaccine coverage: Results of two years of the portal VaccinarSì. Epidemiol. Prev..

[71] Costantino C., Caracci F., Brandi M., Bono S.E., Ferro A., Sannasardo C.E., Scarpitta F., Siddu A., Vella C., Ventura G. (2020). Determinants of vaccine hesitancy and effectiveness of vaccination counseling interventions among a sample of the general population in Palermo, Italy. Hum. Vaccin. Immunother..

[72] World Health Organization (WHO) Barriers of Influenza Vaccination Intention and Behavior—A Systematic Review of Influenza Vaccine Hesitancy 2005–2016. https://apps.who.int/iris/bitstream/handle/10665/251671/WHO-HIS-TTi-GAP-16.2-eng.pdf?sequence=1&isAllowed=y.

[73] Crocker-Buque T., Mindra G., Duncan R., Mounier-Jack S. (2017). Immunization, urbanization and slums—A systematic review of factors and interventions. BMC Public Health.

[74] O’Leary S.T., Barnard J., Lockhart S., Kolasa M., Shmueli D., Dickinson L.M., Kile D., Dibert E., Kempe A. (2015). Urban and rural differences in parental attitudes about influenza vaccination and vaccine delivery models. J. Rural Health.

[75] Restivo V., Orsi A., Ciampini S., Messano G.A., Trucchi C., Ventura G., Casuccio A., Vitale F. (2019). How should vaccination services be planned, organized, and managed? Results from a survey on the Italian vaccination services. Ann. Ig.

[76] Odone A., Lugo A., Amerio A., Borroni E., Bosetti C., Carreras G., Cavalieri d’Oro L., Colombo P., Fanucchi T., Ghislandi S. (2020). COVID-19 lockdown impact on lifestyle habits of Italian adults. Acta Biomed..

[77] Vukovic V., Lillini R., Lupi S., Fortunato F., Cicconi M., Matteo G., Arata L., Amicizia D., Boccalini S., Bechini A. (2020). Identifying people at risk for influenza with low vaccine uptake based on deprivation status: A systematic review. Eur. J. Public Health.

[78] Lucyk K., Simmonds K.A., Lorenzetti D.L., Drews S.J., Svenson L.W., Russell M.L. (2019). The association between influenza vaccination and socioeconomic status in high income countries varies by the measure used: A systematic review. BMC Med. Res. Methodol..

[79] Rizzo C., Rota M.C., Bella A., Giannitelli S., De Santis S., Nacca G., Pompa M.G., Vellucci L., Salmaso S., Declich S. (2010). Response to the 2009 influenza A(H1N1) pandemic in Italy. Eur. Surveill..

[80] Li Q., Tang B., Bragazzi N.L., Xiao Y., Wu J. (2020). Modeling the impact of mass influenza vaccination and public health interventions on COVID-19 epidemics with limited detection capability. Math. Biosci..

[81] Italian National Institute of Statistics (ISTAT) Internet: Access and Usage Type. http://dati.istat.it/Index.aspx?DataSetCode=DCCV_ICT.

[82] Poynton T.A., DeFouw E.R., Morizio L.J. (2019). A systematic review of online response rates in four counseling journals. J Couns. Dev..

[83] World Health Organization (WHO) The Guide to Tailoring Immunization Programmes (TIP). https://www.euro.who.int/__data/assets/pdf_file/0003/187347/The-Guide-to-Tailoring-Immunization-Programmes-TIP.pdf.

[84] Costantino C., Restivo V., Tramuto F., Casuccio A., Vitale F. (2019). Influenza vaccination of healthcare workers in Italy: Could mandatory vaccination be a solution to protect patients?. Future Microbiol..

