# Supplementary material for: Attitudes and Beliefs on Influenza Vaccination during the COVID-19 Pandemic: Results from a Representative Italian Survey
Source: Vaccines (Basel). 2020 Nov 30;8(4):711. doi: 10.3390/vaccines8040711 (PMC7712959; doi:10.3390/vaccines8040711)
Supplement: Supplementary file 1 [file vaccines-08-00711-s001.pdf]

Table S1. Multivariable logistic regression analysis on the association between socioeconomic factors and reluctance to receive the 2020/21 influenza vaccine: sensitivity analysis (N = 2,262).

Variable	Level	aOR (95% CI)	*P*
Sex	Male	Ref	–
	Female	0.96 (0.79–1.18)	.72
Age, years	1-year increase	0.98 (0.97–0.99)	< .001
2019/20 vaccination	Yes	Ref	–
	No	0.11 (0.08–0.14)	< .001
Geographic area	North-East	Ref	–
	North-West	1.11 (0.83–1.48)	.48
	Center	1.00 (0.73–1.37)	.98
	South	0.79 (0.58–1.08)	.14
	Islands	1.03 (0.71–1.49)	.88
Urbanization pattern, *N* of inhabitants	< 5,000	Ref	–
	5,000–9,999	0.92 (0.63–1.36)	.69
	10,000–29,999	0.61 (0.43–0.87)	.006
	30,000–99,999	0.71 (0.50–0.99)	.049
	100,000–249,999	0.77 (0.51–1.15)	.20
	≥ 250,000	0.56 (0.40–0.79)	< .001
Employment status	Permanently employed	Ref	–
	Occasionally employed	0.78 (0.54–1.15)	.21
	Student	0.52 (0.35–0.77)	.001
	Housekeeper	0.86 (0.55–1.33)	.49
	Unemployed	1.27 (0.89–1.81)	.19
	Retired	0.83 (0.56–1.22)	.33
	Other	0.66 (0.32–1.34)	.25
Household pattern	Living alone	1.32 (0.92–1.90)	.13
	Living with a spouse/partner	1.04 (0.83–1.31)	.73
	Living with ≥ 1 child	1.21 (0.97–1.50)	.092
	Living with ≥ 1 older adult	1.05 (0.65–1.69)	.85
Perceived economic well-being	Low	Ref	–
	Lower than average	0.60 (0.32–1.12)	.11
	Average	0.49 (0.28–0.87)	.016
	Higher than average	0.47 (0.26–0.83)	.010
	High	0.33 (0.15–0.72)	.006
ISCED educational level	1	Ref	–
	2	0.54 (0.15–1.94)	.34
	3/4	0.65 (0.19–2.23)	.49
	5	0.52 (0.15–1.94)	.31
	6	0.63 (0.19–2.23)	.54
